# Comparison of Different Antibiotic Regimes for Preventive Tooth Extractions in Patients with Antiresorptive Intake—A Retrospective Cohort Study

**DOI:** 10.3390/antibiotics12060997

**Published:** 2023-06-01

**Authors:** Oliver Ristow, Thomas Rückschloß, Gregor Schnug, Julius Moratin, Moritz Bleymehl, Sven Zittel, Maximilian Pilz, Caroline Sekundo, Christian Mertens, Michael Engel, Jürgen Hoffmann, Maximilian Smielowski

**Affiliations:** 1 Department of Oral and Maxillofacial Surgery, University of Heidelberg, Im Neuenheimer Feld 400, D-69120 Heidelberg, Germany; 2 Department of Biometry, Institute of Medical Biometry, University of Heidelberg, Im Neuenheimer Feld 130.3, D-69120 Heidelberg, Germany; 3 Department of Conservative Dentistry, University of Heidelberg, Im Neuenheimer Feld 400, D-69120 Heidelberg, Germany

**Keywords:** MRONJ, BRONJ, prevention, risk reduction, tooth extraction, antibiotics

## Abstract

In the present study, the impacts on success rates between three different antibiotic regimes in patients receiving preventive tooth extraction during/after antiresorptive treatment were compared. For the retrospective analysis, we enrolled patients who had undergone tooth extraction from 2009 to 2019 according to the specified preventive conditions under antiresorptive therapy. Three antibiotic regimens were distinguished: (Group 1) intravenous for 7 days, (Group 2) oral for 14 days, and (Group 3) oral for 7 days of application. The primary endpoint was the occurrence of medication-related osteonecrosis of the jaw at 12 weeks after surgery. A total of 760 patients and 1143 extraction regions were evaluated (Group 1 *n* = 719; Group 2 *n* = 126; Group 3 *n* = 298). The primary endpoint showed no significant difference in the development of medication-related osteonecrosis of the jaw between the groups studied (Group 1 *n* = 50/669 (7%); Group 2 *n* = 9/117 (7%); Group 3 *n* = 17/281 (6%); *p* = 0.746). Overall, the success rate was 93% after intervention when preventive measures were followed. With the same success rate, a reduced, oral administration of antibiotics seems to be sufficient regarding the possible spectrum of side effects, the development of resistance and the health economic point of view.

## 1. Introduction

With better knowledge of the problems involved, minimization of the risk of medication-related osteonecrosis of the jaw (MRONJ) in patients receiving antiresorptive (AR) therapy is becoming increasingly relevant [1,2,3,4]. The time window of prevention (defined as risk reduction during/after AR therapy) is especially important, not only because the most uncertainty about the optimal course of action occurs during this period, but also because a differential diagnostic challenge between preventive measures must be faced. Such diagnostics are necessary because of the pathology originating from the dentoalveolar system and the need for the early detection of manifest osteonecrosis [5,6,7]. A search for the literature concerning this time window quickly reveals disagreements in the risk reduction measures that are necessary [8]. This is mainly because tooth extractions are still believed to be the major risk factor for the development of MRONJ [9,10,11,12,13,14]. Thus, even the latest update from the American Association of Oral and Maxillofacial Surgeons (AAOMS) still recommends avoiding procedures that involve direct osseus injury for patients receiving AR therapy for cancer [15]. More and more data indicate that tooth extraction alone is not responsible for the development of MRONJ, but that the infection leading to tooth extraction often triggers the pathogenesis cascade [16,17,18,19]. On the assumption that these data are reliable, any decision to leave inflammatory lesions in place for fear of provoking osteonecrosis during surgery would therefore be wrong. The latter theory is supported by numerous data reporting high rates of early nonvital changes in alveolar bone structure in the area of direct contact with teeth at the time of tooth extraction [20,21,22,23,24]. It is also important to recognize that early necrotic lesions are found with different frequency rates but in all risk groups and thus must be considered as potential predilection sites for MRONJ [21,22]. Based on this suggestion, many international working groups have been able to demonstrate that successful tooth extractions during/after AR therapy with low MRONJ incidences are possible, provided that the following preventive measures are undertaken [17,25,26]: (1) antibiotic therapy, (2) alveoplasty, and (3) primary wound closure. All these measures are performed to eliminate bacterial contamination, infection, and/or early non-vital bone changes with direct contact to the teeth [27,28]. Knowledge that teeth can be safely removed, even under ongoing AR therapy and regardless of the risk group the patient belongs to, brings several advantages: First, in the sense of the infection-dependent pathogenesis theory, potential predilection foci can be removed promptly if dental preservative therapy appears futile. Second, before AR therapy is started, focused rehabilitation is recommended to minimize the risk of MRONJ over time, although urgently needed AR therapy should never be postponed because of a potential dental focus. The knowledge that teeth can also be safely removed during/after ongoing AR therapy thus has a positive influence on the risk–benefit constellation and in cases in which a timely start of AR therapy is necessary. Finally, the knowledge that teeth can be safely removed, even under ongoing AR therapy, helps to reduce patient and practitioner anxiety.

However, no clear consensus has yet been achieved regarding the choice and execution of risk reduction measurements [1,8,29,30]. As there are no striking data, several studies showed divergent perception leading to disputes and causing inconstant success rates in MRONJ from incidences as low as 3% up to 43% following preventive tooth extractions [31,32]. Clearly, any success is derived from the total individual risk reduction measures as listed above. Good data now exist for the legitimacy of alveoplasty and primary wound closure [21,22,28,32]. However, little information is available for the best-known and most widely used measure, namely the concomitant administration of antibiotics. Variation is found not only in the choice of the antibiotic spectrum to be prescribed and the means of its administration and dosage, but also (and to a greater extent) in the duration of its administration [22,33,34,35].

In the literature, antibiotic administration ranges from long-term administration before surgical tooth extraction in order to achieve sufficient drug coverage of the bone to exclusively short-term perioperative administrations and to long-term prolonged administration up to 30 days after tooth removal [20,22,33,36]. In view of the known side effects of antibiotics, the increasing development of resistance to certain strains in a mostly immunosuppressed patient population, and the increasing costs to the health care system, the aim should be to reduce the number of antibiotics administered to a minimum and, above all, to minimalize the duration of administration to as much as absolutely necessary [37]. To the best of our knowledge, however, no studies to date have examined the importance of various antibiotic regimens in the overall context of risk reduction measures in patients during/after AR therapy.

Therefore, the purpose of this study is to determine whether differing antibiotic regimes have an impact on the treatment outcome after surgical tooth extractions in high- and low-risk patients during or after undergoing AR treatment. We hypothesize that the choice of antibiotic regime does not influence the therapy success rate providing that the other risk reduction measures (alveoplasty and primary wound closure) are also carried out.

## 2. Results

### 2.1. Study Sample

A total of 759 patients were included with 1143 treated extraction sites and 2010 removed teeth. A total of 71% (540/759) patients were female and 29% (219/759) were male. Patients with underlying diseases were divided as follows: breast carcinoma, 31% (238/759); prostate carcinoma, 7% (52/759); multiple myeloma, 16% (118/759); other malignant disease, 6% (44/759); and osteoporosis, 40% (307/759).

According to their antibiotic regime, of the overall 1143 treated extraction sites, 719 (63%) were distributed to Group 1 (intravenous AB), 298 (26%) to Group 2 (oral AB 14 days), and 126 (11%) to Group 3 (oral AB for 7 days). AB therapy was mainly performed with beta lactam AB. Clindamycin (132 cases, 12%) and other moxifloxacin (40 cases, 3%) were only applied rarely. The distribution between intervention groups was balanced (see also Table 1).

### 2.2. Primary Endpoint

Across all groups, at 3 months after surgery, complete mucosal recovery was achieved in 93% (1067/1143) of all extraction sites, whereas treatment failed and MRONJ developed in 7% (76/1143) of the cases. Stratified for risk groups, complete mucosal recovery was achieved in 90% (594/660) of the cases in the high-risk group and in 98% (473/483) of the cases in the low-risk group.

No statistically significant difference in therapy success was seen between the three different intervention groups, namely the antibiotic regimes. In Group 1 (intravenous AB for 7 days), therapy success was achieved in 93.0% (669/719), failure in 7.0% (50/719) of the cases. For Group 2 (oral AB for 14 days), closed mucosa was achieved in 94.3% (281/298) of the cases at 3 months after surgery, whereas 5.7% (17/298) of the patients developed MRONJ. In Group 3 (oral AB for 7 days), therapy success was achieved in 92.9% (117/126) of the cases, and 7.1% (9/126) of the patients developed MRONJ at the primary endpoint. Comparison of Groups 1 and 2 yielded an odds ratio of 0.77 (95%CI: [0.37; 1.61], *p* = 0.485), comparison of Groups 1 and 3—an odds ratio of 1.17 (95%CI: [0.44; 3.12], *p* = 0.755), and comparison of Groups 3 and 2—an odds ratio of 0.66 (95%CI: [0.22; 1.99]; *p* = 0.458). Therefore, no statistical significance was observed.

### 2.3. Predictive Variables

An overview of the exploratory analysis of predictive variables influencing the outcome of surgical success, which was defined as complete mucosal recovery and freedom from symptoms, is presented in Table 2. The assignment to the groups of high-risk patients (OR: 0.15, 95%CI: [0.07; 0.35], *p* = 8.3 × 10^−6^) and the preventive extractions performed in the lower jaw (OR: 1.85, 95%CI: [1.03; 3.33], *p* = 0.0391) had a statistically significant negative effect on the therapeutic success (complete mucosal recovery after 3 months).

## 3. Discussion

The purpose of this study was to determine whether differing antibiotic regimes had an impact on treatment outcome after surgical tooth extractions in high- and low-risk patients during or after the AR treatment. The authors hypothesized that the choice of antibiotic regime would not influence the therapy success rate provided that the other risk reduction measures (alveoplasty and primary wound closure) were carried out. The results of this study showed that the risk of developing MRONJ after a preventive tooth extraction was low in all patients at around 7% provided that all risk reduction conditions were thoroughly met. The choice of antibiotic regimen had no significant influence on the success rate. Negative predictive influence on the therapeutic success was allocated to the high-risk group, as were extractions in the mandible. Interestingly, patients with simultaneous osteonecrosis in another quadrant (not relevant to the preventive extraction site) did not have higher MRONJ event rates than patients without pre-existing necrosis.

Historically, antibiotic prophylaxis has been the most accepted risk reduction measure in tooth extractions during/after AR therapy. Overall, the data quality on which the recommendation for antibiotic adjunctive therapy is based is limited [33]. However, this approach is based on the idea of reducing infection and bacterial colonization of the extraction socket and thus is comprehensible in the sense of MRONJ’s infection-related pathogenesis theory [16,19]. Most guideline recommendations are based on Montefusco et al., one of the few studies that has addressed this issue [38]. The authors conducted a retrospective study of multiple myeloma patients to determine whether antibiotic prophylaxis prior to dental procedures relevantly reduced their risk of developing MRONJ. Of the 75 patients meeting the criteria, 43 were allocated to the antibiotic group and 32 to the no antibiotic group. Eight patients developed MRONJ, of whom all were from the no antibiotic group (*p* = 0.012). The authors concluded that antibiotic prophylaxis reduced the incidence of ONJ after dental procedures. Although the publication of Montefusco et al. represents a milestone in the recommendation of prophylactic antibiotic delivery in AR patients, the data must be interpreted carefully. The dental procedures reported in the protocol are inhomogeneous since they are not limited to tooth extractions, ranging from professional tooth cleaning to pre-prosthetic surgery.

In a recent prospective study, Park et al. included all patients (*n* = 76) with an indication for dentoalveolar surgery after AR therapy and reported a high incidence of 43% MRONJ after intervention but without having applied antibiotics or any other risk reduction measures [31]. Of note, the authors included both high- (17/76) and low-risk (59/76) patients in their evaluation, and, like Montefusco et al., combined data from tooth extractions (52/76) with other dentoalveolar surgical procedures involving surgical trauma to the jawbone (24/76), thereby reducing the comparability of the results. To counteract such confounding factors, we attempted to narrow the inclusion criteria in this study and focused only on preventive tooth extractions to obtain a more homogeneous study population.

Overall, in the literature, the success rates after preventive tooth extractions involving antibiotics are heterogeneous, with comparisons in some studies showing over a 40% difference in success [12,20,21,22,32,39,40,41,42,43,44]. One of the reasons for this wide range is probably the consideration of different risk groups. Thus, even our study has found a negative predictive value of 10% MRONJ rates if the patients belong to the high-risk group (i.e., monthly high-dose AR therapy) compared with 2% MRONJ rates in the low-risk group. Care must be taken in the epidemiological classification of risk groups. As in the latest guideline update of the AAOMS, which is still considered the gold standard for many guideline groups, allocation is usually based on the patient’s underlying disease [15]. Thus, all patients with osteoporosis are grouped into the low-risk group, and all patients with underlying malignancy are grouped into the high-risk group. Based on this allocation, guidelines often primarily decide not only which preventive, but also which therapeutic algorithms will be applied [2,13,15,17,25,45]. This approach is misguided, both epidemiologically and clinically, for several reasons. Increasing data show that certain risk factors have an impact on the patient-specific risk profile independent of the underlying disease. In simple summary, risk increases with dosage, duration of antiresorptive therapy, concomitant immunomodulatory diseases and medications, and local risk factors (inflammation in the oral cavity). A simple assignment into high- and low-risk groups based on an underlying disease becomes erroneous when this knowledge is taken into consideration. For example, the number of osteoporosis patients who have received antiresorptive treatment for many years and/or have experienced concomitant immunosuppression caused by medication (e.g., chemotherapy, cortisone therapy) or have an immunomodulatory concomitant disease (e.g., diabetes mellitus) has been underestimated. This error might lead not only to misguided recommendations, but also to practitioners underestimating the situation. However, patients with an underlying malignancy who do not receive a significantly higher drug dose because of metastasis to bone but receive adjuvant antiresorptive therapy to prevent or treat secondary osteoporosis are often overestimated [46,47,48].

To counteract this confounding factor, we defined our inclusion and exclusion criteria such that the high-risk group included only those patients who were included because of bony metastasis (or bony metastasis of multiple myeloma), and the low-risk group included only patients with primary osteoporosis. The group of patients with adjuvant therapy was omitted from this study because of the large number of concomitant therapies of the underlying disease as a confounding factor. In future study protocols, this increasingly important group must be considered. However, our allocation also leaves room for criticism. Because of the retrospective nature of our study, concomitant immunosuppression from medication or an immunomodulatory concomitant disease were not considered and reported. In addition, we combined data from patients with multiple myeloma and solid bone metastases, a further possible point of criticism. Interestingly, in contrast to other studies [49,50], the predictive value, duration of intake, has been shown not to have a negative influence on primary outcome in our study.

Nevertheless, a large difference still occurs in success rates when stratifying the literature by tooth extraction in high-risk patients and searching for those who have used the risk reduction measure of antibiosis. For example, Nicolatou-Galitis et al. performed a prospective evaluation on 54 cancer patients who received tooth extraction after AR therapy. Extractions were performed under antibiotic treatment (amoxicillin and metronidazole, three times daily) initiated 2 days prior to and continued up to 30 days after the extraction. The authors reported a high incidence of 26% MRONJ cases at 8 weeks after surgery [20]. The marked difference from the 10% MRONJ event rates in the high-risk group in the present study requires closer consideration. Nicolatou-Galitis et al. did not apply the other preventive measures (alveoplasty and primary wound closure) that we used and that have previously been shown to be of high importance in a number of other studies [21,22,36]. Moreover, this difference can also be seen in the existing literature concerning preventive tooth extractions. The investigations that did not involve any of the recommended preventive measures resulted in the worst outcomes. The studies in which only antibiosis was used as a preventive measure were inferior to those in which two of the preventive measures were employed, whereas studies with all three measures were the most successful [20,21,22,33,39,40,42]. In a recent systematic review that addressed the question of whether peri-procedural administration of systemic antibiotics reduced the risk of MRONJ after preventive tooth extraction, Cabral et al. conclude that, in particular, the additional preventive measures (alveolplasty and primary wound closure) had a positive impact on event rates for all the 17 included studies and reduced the MRONJ frequency to 0–13% versus 5–57% for high-risk patients and 0.1% versus 1% for all low-risk patients [33].

Having established the importance of the two additional preventive measures, we need to take a closer look at the role of antibiotic administration. The basic idea behind the preventive antibiosis used here is that the bone is protected by the antibiosis at the time of exposure to the bacterial flora of the mouth and to the healing process after tooth extraction [33,34]. The question of the ideal period and way of application of the antibiotic before and after surgery is yet to be answered. Optimal bone and soft tissue concentrations as well as broad activity spectrum antibiotic from the time of incision until a competent closure of the surgical incisions can be assumed should be the objectives here. By these means, the oral bacterial flora can be obstructed from openly entering the surgical site. The data regarding these aspects are sparse because the study populations are usually too heterogeneous, or the diverse inclusion and exclusion criteria of the various studies are difficult to compare [22,33,34]. Poxleitner et al. correctly state that the effectiveness of antibiotic shielding depends significantly on the penetration capacity of the antibiotic, and here, bone is the limiting tissue. The above-mentioned authors prospectively investigated the penicillin G concentration in serum and bone samples from 19 MRONJ patients who had received a single dose of intravenous penicillin G prior to surgical therapy [34]. They found that the serum activity of the drug was adequate in all patients, and that bone activity was adequate in 16 of 19 samples after a single administration. They concluded that a short-term administration before intervention in the sense of perioperative shielding is sufficient. Accordingly, antibiotic ablation undertaken with enough time before a surgical intervention makes sense. A distinction must certainly be made here as to whether directly available intravenous administration is possible, or whether oral administration should be used with the necessary lead time. Poxleitner et al. point out that no data exist concerning oral administration and the accumulation of antibiotics in AR therapy patients [34]. Thus, we can deduce that, irrespective of the regimen used, an antibiotic application should take place at least one day before a surgical intervention in order to achieve a sufficient accumulation of antibiotic in the tissue, at least until better data are available.

Furthermore, the duration of postoperative prolonged administration is unclear. The protocol shown here has been adapted from missing evidence based on the following assumptions. A broad activity spectrum is expected approximately after 3–4 days of antibiotic application (for beta-lactam penicillin). Furthermore, after 4 days, a sufficient closure of the superficial surgical incision can be expected, prohibiting oral bacterial flora from freely entering the surgical site [51,52]. This strengthens the assumption that, when prolonged antibiotic administration is carried out, it should be continued at least until a safe accumulation is ensured and bacterial contamination has been interrupted by wound healing. A comparison of our three study groups supports these findings. First, the influence on the primary outcome is no different irrespective of whether antibiotic is administered for a long time (Group 2) or only for a short time (Group 3) before surgery. Furthermore, the data indicate no differences as to whether the antibiotic was delivered intravenously (Group 1) or orally (Group 2 and 3). Further studies with clinical and microbiological data will be necessary in the future to investigate the optimal duration of application.

Indeed, a difference might arise if the teeth to be extracted are symptomatic with infection or asymptomatic. In symptomatic teeth with signs of infection, the role of antibiotic administration might change from concomitant prevention to therapy, and prolonged administration may be necessary [19,21,22,23,37]. The existing data in the literature on preventive tooth extractions under AR therapy must be interpreted carefully in this regard, as the differentiation between infected and non-infected extraction regions has received attention in only a few studies. However, this finding is of great importance, both for physicians and from an epidemiological point of view, since the risk profile changes significantly as a result of acute infection [22,33]. In addition, acutely infected regions are more difficult to differentiate from pre-existing MRONJ [7,53,54]. This point must always be considered when interpreting data, as it directly influences the approach and results. To maintain comparability in this study, we have therefore excluded acutely infected teeth or the suspicion or presence of pre-existing necrosis.

As mentioned above, no evidence has been obtained and, therefore, no consensus has been reached regarding the best choice of antibiotic. In the current study, beta-lactam penicillin was chosen, if no restrictions were indicated. On the one hand, beta-lactam penicillin has become established in the therapy of MRONJ with good coverage of the specific microbiological species often associated with MRONJ (e.g., actinomycetes) [55]. Furthermore, as a standardized antibiotic for oral and head- and neck-associated infections [56], it fulfills the inhibitory concentrations for bacteria, usually part of the oral flora with a tolerable spectrum of side effects. The principal idea of the antibiotic algorithm applied here has therefore been to provide a broad treatment, but only briefly, with the aim of administering as much as necessary and as little as possible, with the long-term goal of minimizing resistance, avoiding side effects, and finally optimizing costs. This choice is consistent with the literature as shown in the recent systematic review by Cabras et al. Of the 17 studies on preventive tooth extraction selected by the authors, 76.4% used penicillin with or without the addition of beta-lactam or metronidazole, 23.5% used clindamycin instead. In line with the core message of the present study, Cabras et al. consider that the concomitant administration of antibiotics in preventive tooth extractions during/after AR therapy is indispensable, although this is based on a purely empirical decision, as the evidence to date is insufficient. The authors conclude that future studies with a combination of clinical and microbiological data will be necessary to establish the main characteristics of a reliable perioperative antibiotic regime [33]. Unfortunately, the retrospective data of our study do not allow conclusions to be drawn about the incident side effects of the various regimens and might be considered as limitation. This problem should be addressed as part of a subsequent prospective study.

To the best of our knowledge, the present study reports one of the largest and homogenous collectives of patients with preventive tooth extraction during/after antiresorptive therapy producing a high external validity. Despite the limitation of the retrospective data acquisition and analysis, a high degree of reliability can be assumed. This is because, on the one hand, all surgical procedures have been performed by the same trained surgeons following a standardized protocol, and because all patients undergoing AR therapy are admitted to our center-specific special consultation and are thus integrated into a long-term follow-up adapted to the underlying disease. This ensures standardized examinations by AR-specialized physicians and a protocol-defined documentation of patient data minimizing bias.

The selected follow-up interval of 3 months might be considered as a limitation of the study protocol. However, this was deliberately chosen on the basis of our earlier studies showing that, if the extraction regions heal after 8 weeks, no MRONJ arises in connection with the surgical procedure, even over a longer period of time [5,22]. Additionally, the 3-month period after surgery is the internationally accepted interval during which surgical site infections can occur and be diagnosed as a relevant outcome in antibiotic studies [57,58,59].

From an intervention perspective, future studies are necessary that critically question the extent of the preventive measures applied. The aim should be to determine those measures that are necessary for a patient within a benefit–risk assessment. This would not only optimize and avoid unnecessary antibiotic administration, but also prevent unnecessary overstress to the preprosthetic bearing (both soft tissue and bony). However, to ensure patient safety, these studies should be conducted in low-risk groups.

## 4. Materials and Methods

### 4.1. Study Design

This study was designed as a confirmatory, retrospective, longitudinal cohort study. Investigations were carried out at in a single center (Department of Oral and Cranio-Maxillofacial Surgery, Heidelberg University Hospital, Heidelberg, Germany). The study was approved by the designated Research Ethic Board (S-514/2020) and conducted in full accordance with ethical principles and the Declaration of Helsinki. The manuscript was prepared according to the STROBE guidelines [60].

### 4.2. Study Sample

All patients that were assigned between 1 January 2009 and 31 December 2019 for teeth removal in accordance with the preventive conditions during/after AR therapy with bisphosphonates or denosumab were included in this analysis. The inclusion criteria were as follows: (a) indication for tooth removal; (b) ongoing or previous history of antiresorptive treatment (bisphosphonate or denosumab), stratified either as high-risk patients (AR therapy according to the protocols for the therapy of malignant disease with bone metastasis or multiple myeloma) or as low-risk patients (AR therapy according to the protocols for the therapy of primary osteoporosis); (c) no signs of exposed or probable bone to the oral cavity or radiological suspicion of ONJ (corresponding to AAOMS stage 0), and therefore no signs of MRONJ. Patients were divided into three groups depending on their predictor variable type of antibiotic regime: Group 1: intravenous antibiotics for 7 days starting 1 day before surgery; Group 2: oral antibiotics for 14 days starting 7 days before extraction; and Group 3: oral antibiotics for 7 days starting 2 days before extraction.

Exclusion criteria were: (a) acutely affected teeth with the need for therapeutic antibiosis, (b) fistula to the teeth, (c) a history of MRONJ at the extraction site, (d) a history of head and neck radiation, (e) known malignant or metastatic bone disease of the maxillofacial region, (f) malignant disease without metastasis treated with AR medication for the therapy of secondary osteoporosis, and (g) an age younger than 18 years.

### 4.3. Study Variables

The predictor variable was the type of antibiotic regime used as a preventive measure during tooth extraction in patients during/after AR treatment. Three different antibiotic regime groups were defined. Group 1 (i.v.) received intravenous antibiotics for one week, Group 2 (two weeks) received oral antibiotics for two weeks and Group 3 (one week) received oral antibiotics for one week. The primary outcome was surgical success at 12 weeks after intervention (success was defined as complete mucosal recovery and freedom from symptoms). Demographics, baseline characteristics, and confounding variables for treatment failure were also collected as listed in Table 1.

### 4.4. Surgical Protocol

All surgical tooth extractions were performed by the same experienced surgeons following the standardized protocol and risk reduction measures for patients undergoing/after AR therapy as previously described by our group and in accordance with the German guideline [5,22,26,54]: (1) adjunctive antibiotic therapy (according to the assigned groups), (2) alveoplasty, and (3) thorough primary wound closure. In the case of purely preventive tooth extraction, the procedure was performed in an outpatient setting under local anesthesia with oral antibiotics. In the case of concomitant therapy for simultaneous MRONJ in another quadrant (i.e., one not relevant for this study), the preventive tooth extraction was performed during necrosis surgery under general anesthesia and in an in-patient setting with the intravenous administration of antibiotics.

In detail, after local anesthesia in the region of interest (Ultracain^®^ D-S 1:200,000, Sanofi-Aventis Deutschland GmbH, 65926 Frankfurt am Main, Germany), intracrevicular incisions were cut in the lateral and medial sulcus of the teeth in need of extraction, with releasing mesial and distal incisions. A muco-periosteal flap was prepared adapted to the needs of the patient in the usual way, followed by the extraction of the teeth in an atraumatic fashion and, if needed, supported by osteotomy. Afterwards, the remaining edges and the floor of the alveolar socket were reduced through alveoplasty completed with thorough levelling of any left bony edges in the area with a round bur. To achieve a tension-free wound closure, periosteal relieving incisions were cut, and all wound margins were stripped of epithelia. Finally, primary wound closure was performed with Vicryl 3-0/4-0 backstitches and single button sutures (VICRYL^®^, Johnson & Johnson Medical N.V., Av. du Port 86C, 1000 Bruxelles, Belgium).

As a first-line drug, beta-lactam antibiotics (Amoclav^®^ 875 mg + 125 mg, Hexal AG, Holzkirchen, Germany, for oral intake, and Unacid^®^ 1.5 g, Pfizer Pharma GmbH, Berlin, Germany, for intravenous intake) were administered to the patients. In patients who reported a history of hypersensitivity to penicillin or a penicillin allergy, clindamycin (Clinda-saar^®^ 600 mg, Chephasaar Chem.-pharm. Fabrik GmbH, St. Ingbert, Germany, for oral intake, and Clindamycin-ratiopharm^®^ 600 mg/4 mL, ratiopharm GmbH, Ulm, Germany, for intravenous intake) was used instead. In rare cases of patients with a history of adverse effects of both beta-lactam antibiotics and clindamycin, another antibiotic was chosen for both intravenous and oral groups.

Furthermore, patients were obliged to use an antiseptic and disinfectant mouthwash and rinse the oral cavity thrice daily from 2 days prior to the procedure up to removal of sutures after 10 to 14 days (Chlorhexamed FORTE alkoholfrei 0.2%, GlaxoSmithKline Consumer Healthcare GmbH & Co. KG, Barthstraße 4, 80339 München, Germany). If the physician in charge of the oncological or osteological treatment of the patient saw no contraindications for a “drug holiday”, antiresorptive medication was discontinued starting at 4 weeks before surgery and lasting until 4 after the procedure.

Patients were scheduled for follow-up examinations in the outpatient department at 10 to 14 days, 4 weeks, and 3 months after the intervention, where MRONJ-treatment-trained investigators conducted clinical checkups.

### 4.5. Statistical Protocol

Statistical analysis was performed with the statistical software R version 4.1.2. Descriptive analysis included the report of absolute and relative frequencies for categorical data and the means and standard deviations for continuous data.

Since multiple cases per patient were observed, mixed logistic regression models with random effect patient were applied to investigate the primary endpoint. The intervention was used as a fixed effect and the surgical success (yes/no) as an independent variable. To obtain odds ratios (OR), two out of the three intervention groups were compared pairwise.

Mixed logistic regression models were applied in order to detect predictive variables in an exploratory analysis, with the surgical success as an independent variable, the respective potential risk factor as a fixed effect, and the patient as a random effect.

ORs are reported together with their 95% confidence interval (CI). Differences are considered statistically significant when the *p*-value is lower than 5% (*p* < 0.05).

## 5. Conclusions

Overall, the MRONJ event rates after preventive tooth extractions can be kept low by adherence to the established measures: (1) antibiotic therapy, (2) alveoplasty, and (3) primary wound closure. The choice of antibiotic regimen does not seem to play a decisive role regarding the preventive removal of non-infected teeth considered unworthy of preservation. In order to minimize side effects and resistance in the long term, the antibiotic regime should therefore be broad and spectrum-adapted and should be undertaken only as long as is necessary. Indeed, differentiation from infected extraction sites is of the utmost importance. In this latter case, a prolonged antibiotic therapy might be inevitable, and an early MRONJ must be excluded by accurate differential diagnosis. The goal should be a risk-adapted and patient-specific preventive concept of therapy that is applied with all involved disciplines closely coordinated.

## Figures and Tables

**Table 1:** Baseline characteristics.

Antibiotic (AB) Regime *	i.v. (*N* = 719)	Two Weeks (*N* = 298)	One Week (*N* = 126)	Total (*N* = 1143)
**gender** female male				
	501 (70%)	213 (71%)	89 (71%)	803 (70%)
	218 (30%)	85 (29%)	37 (29%)	340 (30%)
**risk group** high-risk low-risk				
	461 (64%)	140 (47%)	59 (47%)	660 (58%)
	258 (36%)	158 (53%)	67 (53%)	483 (42%)
**antiresorptive therapy** bisphosphonates denosumab both				
	514 (71%)	217 (73%)	89 (71%)	820 (72%)
	142 (20%)	62 (21%)	23 (18%)	227 (20%)
	63 (9%)	19 (6%)	14 (11%)	96 (8%)
**period of application** (**months**) mean ±sd				
	50	47	47	49
	42	46	46	43
**AB group** beta-lactam clindamycin other				
	612 (85%)	252 (85%)	107 (85%)	971 (85%)
	85 (12%)	38 (13%)	9 (7%)	132 (12%)
	22 (3%)	8 (3%)	10 (8%)	40 (3%)
**site** upper jaw lower jaw				
	351 (49%)	148 (50%)	66 (52%)	565 (49%)
	368 (51%)	150 (50%)	60 (48%)	578 (51%)
**mucosae** closed open				
	669 (93%)	281 (94%)	117 (93%)	1067 (93%)
	50 (7%)	17 (6%)	9 (7%)	76 (7%)

* Demographics and baseline characteristics for group comparability (i.v., AB = Group 1, two weeks AB = Group 2, one week AB = Group 3, and total) shown as numbers of treated extraction sites. Period of application, mean duration, and standard deviation are given in months of application of AR drug.

**Table 2:** Predictive variables.

Mucosae	Closed (*N* = 1067)	Open (*N* = 76)	Total (*N* = 1143)
**gender** female male			
	756 (71%)	47 (62%)	803 (70%)
	311 (29%)	29 (38%)	340 (30%)
risk group * high-risk low-risk			
	594 (56%)	66 (87%)	660 (58%)
	473 (44%)	10 (13%)	483 (42%)
**antiresorptive therapy** bisphosphonates both denosumab			
	767 (72%)	53 (70%)	820 (72%)
	89 (8%)	7 (9%)	96 (8%)
	211 (20%)	16 (21%)	227 (20%)
**period of application** (**months**) mean ±sd			
	49	47	49
	43	44	43
AB therapy ** i.v. one week two weeks			
	669 (63%)	50 (66%)	719 (63%)
	117 (11%)	9 (12%)	126 (11%)
	281 (26%)	17 (22%)	298 (26%)
**AB type** beta-lactam clindamycin other			
	907 (85%)	64 (84%)	971 (85%)
	122 (11%)	10 (13%)	132 (12%)
	38 (4%)	2 (3%)	40 (3%)
site *** upper jaw lower jaw			
	536 (50%)	29 (38%)	565 (49%)
	531 (50%)	47 (62%)	578 (51%)

Results for mucosal integrity at primary endpoint shown as total numbers of treated extraction sites. (*) The low-risk group showed mucosal integrity statistically significantly more often than the high-risk group (*p* < 0.001). No statistical superiority was apparent for any of the three AB regimes (**). Extractions in the lower jaw (***) were responsible for the majority of treatment failures with statistical significance (*p* < 0.042).

## Data Availability

The data presented in this study are available in justified cases on request from the corresponding author.
